# Understanding the diagnosis of superior mesenteric artery syndrome: analysis of the location of duodenal impression on upper gastrointestinal studies

**DOI:** 10.1007/s00247-023-05782-8

**Published:** 2023-10-14

**Authors:** Scott Caterine, Nikhil S. Patil, Heba Takrouri, Robert M. Issenman, Nina R. Stein, John Donnellan, Ali Yikilmaz

**Affiliations:** 1 Department of Pediatric Radiology, McMaster Children’s Hospital, Hamilton, ON Canada; 2 Michael G. Degroote School of Medicine, McMaster University, Hamilton, ON Canada; 3 Department of Pediatric Gastroenterology, McMaster Children’s Hospital, Hamilton, ON Canada; 4 Diagnostic Imaging, Hamilton Health Sciences, McMaster Children’s Hospital, Room 2S28, 1200 Main St. West, Hamilton, ON L8N 3Z5 Canada

**Keywords:** Children, Duodenal obstruction, Duodenum, Fluoroscopy, Superior mesenteric artery syndrome, Upper gastrointestinal series

## Abstract

**Background:**

Upper gastrointestinal (GI) contrast studies are frequently requested to aid superior mesenteric artery syndrome diagnosis, a rare entity. Compression of the third duodenal part is expected to be mid-to-left of the midline where the superior mesenteric artery arises from the aorta; however, a duodenal impression to the right of the midline due to normal anatomic impression by the inferior vena cava (IVC) is often encountered and frequently misdiagnosed.

**Objective:**

The purpose of this study was to determine the frequencies of (1) normal right-of-midline duodenal impressions and (2) mid-to-left of midline compressions in upper GI studies in a tertiary pediatric referral center.

**Materials and methods:**

All upper GI studies performed at our institution over 2 years were retrospectively evaluated to determine whether the duodenum had vertical duodenal impression to the right of the vertebral midline, mid-to-left of the vertebral midline, or no identifiable duodenal impression at all.

**Results:**

In total, 538 upper GI studies were included in this analysis. A total of 275 male and 247 female patients between 0 and 17 years of age (median: 6 years, range: 1 month-17 years) were included. Of 538 total upper GI studies, there were 240 studies (44.6%) with a right-of-midline impression. There were only 10 studies (1.9%) with a mid-to-left of midline compression, and 9/10 also showed a concurrent right-sided impression sign.

**Conclusion:**

Right-of-midline duodenal impression is a normal anatomic finding caused by the IVC and should not be confused with superior mesenteric artery syndrome. In the presence of an appropriate clinical context, proximal duodenal dilation, “to-and-fro” motion of contrast, and duodenal impression at mid-to-left of midline, a diagnosis of superior mesenteric artery syndrome should be considered.

**Graphical Abstract:**

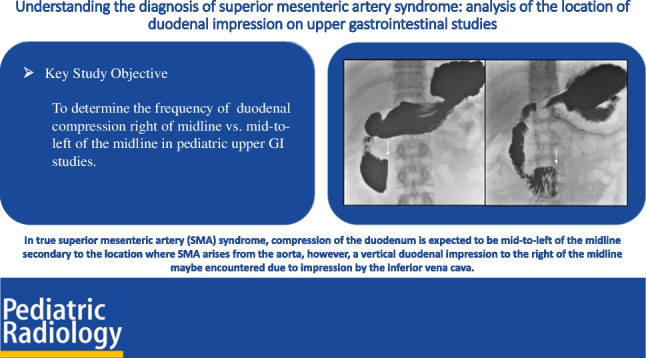

## Introduction

Superior mesenteric artery syndrome is a rare entity characterized by bowel transit impediment secondary to compression of the third part of the duodenum (D3) by the superior mesenteric artery anteriorly and the aorta or vertebrae posteriorly [1–3]. This compression classically leads to nonspecific symptoms including nausea, vomiting, abdominal pain, anorexia, abdominal distension, and weight loss [1, 4–6]. It is important to identify superior mesenteric artery syndrome as it can lead to dehydration, metabolic imbalance, and in rare cases, death [6–8].

**Fig. 1:**
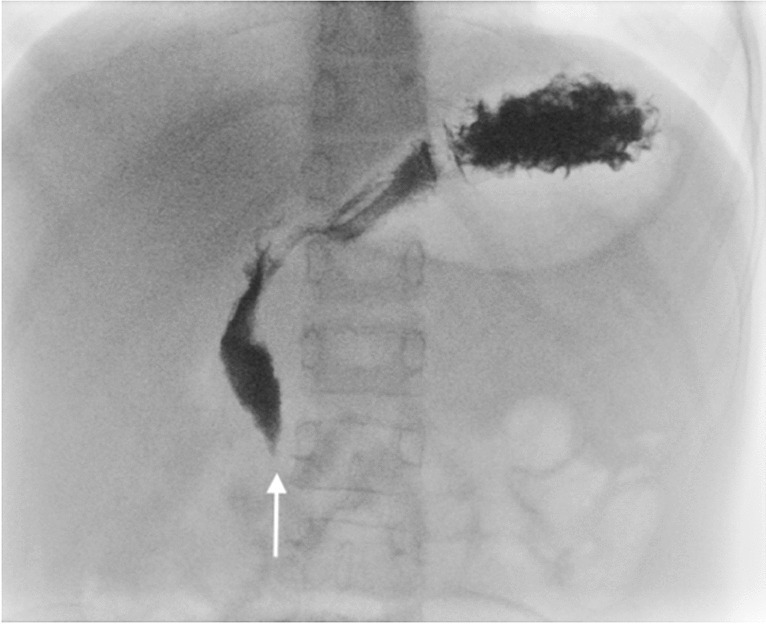
Upper GI contrast study of a 6-year-old male with dysphagia and difficulty swallowing demonstrates a vertical impression to the right-of-midline (*arrow*) with no dilatation of the proximal duodenum or hold-up of contrast. This is a normal finding

**Fig. 2:**
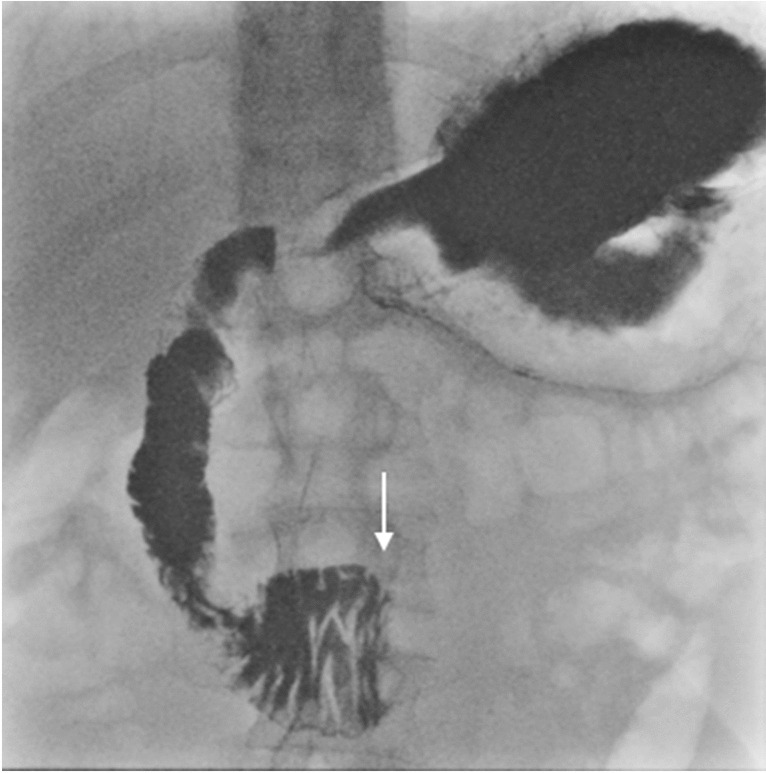
Upper GI contrast study of a 17-year-old male presenting with abdominal pain, emesis, and weight loss. This demonstrates midline compression of the 3rd part of the duodenum in the mid-to-left of midline (*arrow*), where it was reported that there was mild contrast hold-up with to-and-fro peristalsis and eventual passage of contrast, and no proximal duodenal dilatation. There was no clinical suspicion of superior mesenteric artery syndrome, and the study was interpreted as normal

The diagnosis of superior mesenteric artery syndrome is made based on clinical and imaging findings, typically including an upper gastrointestinal (GI) contrast study. Demonstration of an abrupt vertical or oblique compression of the duodenum is one of the hallmark signs in upper GI contrast studies. However, there is often an interobserver discrepancy in the interpretation of this finding. This vertical compression is not always located in the same position on upper GI contrast studies where the superior mesenteric artery is located [9] and resultantly the compression used to diagnose superior mesenteric artery syndrome depicted in the literature is either (1) a vertical duodenal impression to the right of the vertebral midline (Fig. 1) [5, 10–19] or (2) a vertical compression reported in the mid-to-left of the vertebral midline (Fig. 2) [6, 20–28].

The definitive diagnosis of superior mesenteric artery syndrome is obtained through surgical exploration. However, most patients suspected to have superior mesenteric artery syndrome are managed conservatively, with the placement of a nasojejunal tube and a liquid diet [5]. Clinical symptomology and upper GI contrast studies are typically used to diagnose children and should be thought of as the primary means of diagnosis [6, 10, 11, 20, 29–33]. When using upper GI contrast studies, there are generally five diagnostic criteria [1, 5, 6, 13, 14, 34–38]:

1. Dilation of the first and second parts of the duodenum
2. Abrupt vertical or oblique midline compression of the 3^rd^ part of the duodenum
3. “To-and-fro” contrast flow proximal to the compression
4. Delay of transit of the contrast into the jejunum of at least 4–6 h
5. Relief of compression and symptoms in a knee to chest or left lateral decubitus position, also known as Haye’s maneuver

These criteria are further described in Table 1. However, our institutional experience is that the first three criteria are mainly used to diagnose radiologic superior mesenteric artery syndrome. Because of this, we adhere to the following terminology when referring to a diagnosis of superior mesenteric artery syndrome:

1. Definitive superior mesenteric artery syndrome diagnosis: based on surgical confirmation
2. Clinical diagnosis of superior mesenteric artery syndrome: a patient is diagnosed with superior mesenteric artery syndrome by the clinical team (as per patient electronic medical record (EMR) notes), which is typically done after excluding other differential diagnoses and following resolution of patient symptoms with placement of a nasojejunal tube/liquid diet
3. Radiologic superior mesenteric artery syndrome: when all three criteria are present on upper GI studies (1) dilation of the first and second parts of the duodenum; (2) abrupt vertical or oblique mid-to-left of midline compression of the third part of the duodenum; and (3) “to-and-fro” flow of contrast proximal to the compression. These findings must be seen in the appropriate clinical setting to provide a diagnosis of superior mesenteric artery syndrome.

**Table 1:** Five generally accepted upper GI radiographic diagnostic criteria for SMA syndrome

Diagnostic criteria	Definition
1. Dilation of the 1^st^ and 2^nd^ parts of the duodenum	Pathological compression of the 3^rd^ part of the duodenum will result in proximal obstruction and dilatation of the 1^st^ and 2^nd^ parts of the duodenum
2. Abrupt vertical or oblique midline compression of the 3^rd^ part of the duodenum	Anatomically, the 3^rd^ part of the duodenum is crossed by the mesenteric root and passes directly posterior to the SMA
3. “To-and-fro” flow of the contrast proximal to the compression	Compression of the 3^rd^ part of the duodenum will act as a barrier to the forward passage of contrast, resulting in retrograde flow of contrast and forward contrast flow due to peristalsis
4. Delay of transit of the contrast into the jejunum of at least 4–6 h	Contrast transit delay of 4–6 h into the jejunum indicates focal prolonged obstruction at the 3^rd^ part of the duodenum, and not obstruction elsewhere or intermittent/positional obstruction
5. Relief of compression and symptoms in a knee to chest or left lateral decubitus position, also known as Haye’s maneuver	This positional maneuver attempts to increase the aortomesenteric angle (knee to chest) or use gravity (left lateral decubitus position) to assist flow of contrast into the jejunum

Some believe all 5 criteria need to be present for the diagnosis of superior mesenteric artery syndrome [5]; however, others will make a diagnosis of superior mesenteric artery syndrome without all the signs being present [29]. Arguably, the most important sign is the abrupt compression of the 3^rd^ part of the duodenum; however, we suspect this is often incorrectly overcalled due to an anatomic impression by the inferior vena cava (IVC) to the right-of-midline.

The purpose of this study is to review upper GI contrast studies performed in pediatric patients for all indications and determine the frequency of radiographic duodenal impression to the right-of-midline and compare this to the frequency of any duodenal compression at the mid-to-left of midline. Finally, we determine if there is a relationship of these findings in individuals with a potential diagnosis of superior mesenteric artery syndrome in our study cohort.

## Materials and methods

### Data collection

This study was approved by the Integrated Research Ethics Board at our institution. A retrospective analysis of clinical data and all upper GI tract contrast studies regardless of indication, which were performed at a tertiary level pediatric hospital, was conducted including all patients examined between January 2017 and December 2018 aged between 0 and 17 years. A total of 662 studies were reviewed; 124 studies were excluded as they did not include appropriate imaging of the duodenum, leaving 538 total studies included in the final review. Of these, 15 patients had 2 upper GI contrast studies and 1 patient had 3 upper GI contrast studies, all of which were included.

### GI contrast study

Barium solution (E-Z-HDR or PolybarR) was used in the upper GI contrast studies as the contrast agent unless there was a risk for aspiration/perforation or concern for obstruction where water-soluble contrast (Omnipaque 240R or Visipaque 270R) was used instead. The principles of the Image Gently campaign of the Society for Pediatric Radiology and Image Gently Alliance were applied [39]. Pulsed fluoroscopy technique and capturing of last-image hold were performed. Routine protocol included a lateral view of the esophagus in the left lateral decubitus position, anteroposterior (AP) view of the esophagus, AP view of the stomach in the supine position, the pylorus/duodenum in the right/right oblique lateral decubitus positions, AP view of the duodenum in the supine position, and AP view of the proximal jejunum in the supine position. Additional views were obtained as determined by the performing radiologist such as cine clips or left lateral decubitus/prone/erect views.

#### Image analysis

All upper GI studies were reviewed by a pediatric radiologist (A.Y, 15 years of experience; or J.D, 12 years of experience) blinded to clinical data, and a final year medical student. Difficult, discordant, or complex cases were flagged to be reviewed by both pediatric radiologists and a final consensus was achieved.

The first three criteria outlined in Table 1 were noted during analysis of the imaging studies, where assessment of to-and-fro motion was determined based on the initial radiology report, as the reading radiologist was responsible for image acquisition and would have the most context in reporting these findings. Relief of compression and symptoms in a knee to chest or left lateral decubitus position, also known as the Haye’s maneuver, was not recorded or included in our analysis as oftentimes this maneuver may have been performed; however, it was not reliably or consistently recorded in our EMR or radiology report. Similarly, as the standard of care does not entail scanning patients over 4–6 h at our institution, the criterion for delay of transit of the contrast into the jejunum of at least 4–6 h was excluded from our analysis. In regard to the criterion of duodenal compression, we distinguished between the following types in our image analysis:

1. A vertical impression at the junction of the D2-D3 parts (inferior duodenal flexure) located to the right of vertebral midline on AP radiograph position, with an impression defined as a visual difference in contrast outline, at odds with expected normal anatomic boundaries and normal peristaltic flow
2. A vertical compression at the D3 part located in the mid-to-left of vertebral midline
3. Presence of two areas of duodenal impression/compression concurrently as defined by (1) and (2)
4. No vertical impression/compressions in the duodenum, with normal passage of contrast through the duodenum

We deemed radiological superior mesenteric artery syndrome as cases in which the first three criteria outlined in Table 1 were all present where the compression was mid-to-left of midline.

## Results

A total of 662 upper GI contrast studies were reviewed, where 124 studies were excluded due to poor visibility of the region of interest. This was mostly due to the duodenum not being in the area of clinical interest during the scan, patient compliance, or other factors. Over the course of 2 years, 522 patients undergoing a total of 538 UGI studies were included in the study. The study group consisted of 275 male and 247 female patients between 0 and 17 years of age (median: 6 years, range: 1 month-17 years). The most common presenting symptoms leading to the imaging requisition were weight loss, reflux, and dysphagia. In total, 297 out of 538 (55.2%) studies did not show any duodenal impression/compression. In 240 studies (44.6%), there was an impression on the duodenum to the right of vertebral midline. There were 10 studies (1.9%) with a mid-to-left of midline compression. Of these, 9/10 showed a concurrent right-of-midline impression. In all the upper GI studies, there were 10 studies (1.9%) with D1/D2 dilatation and there were 21 studies (3.9%) with “to-and-fro” contrast flow. Only three studies (0.06%) met our three diagnostic criteria for radiologic superior mesenteric artery syndrome, none of whom had a clinical diagnosis of superior mesenteric artery syndrome and thus were not treated as superior mesenteric artery syndrome cases from a clinical perspective. In total, only 15 cases (2.8%) had “persistent” contrast hold-up, defined by contrast hold-up longer than a couple of minutes. Of these 15 cases, 10 had only a right-of-midline duodenal impression, 4 had a right-of-midline impression and mid-to-left of midline duodenal compression, and one had no duodenal impression findings.

**Fig. 3:**
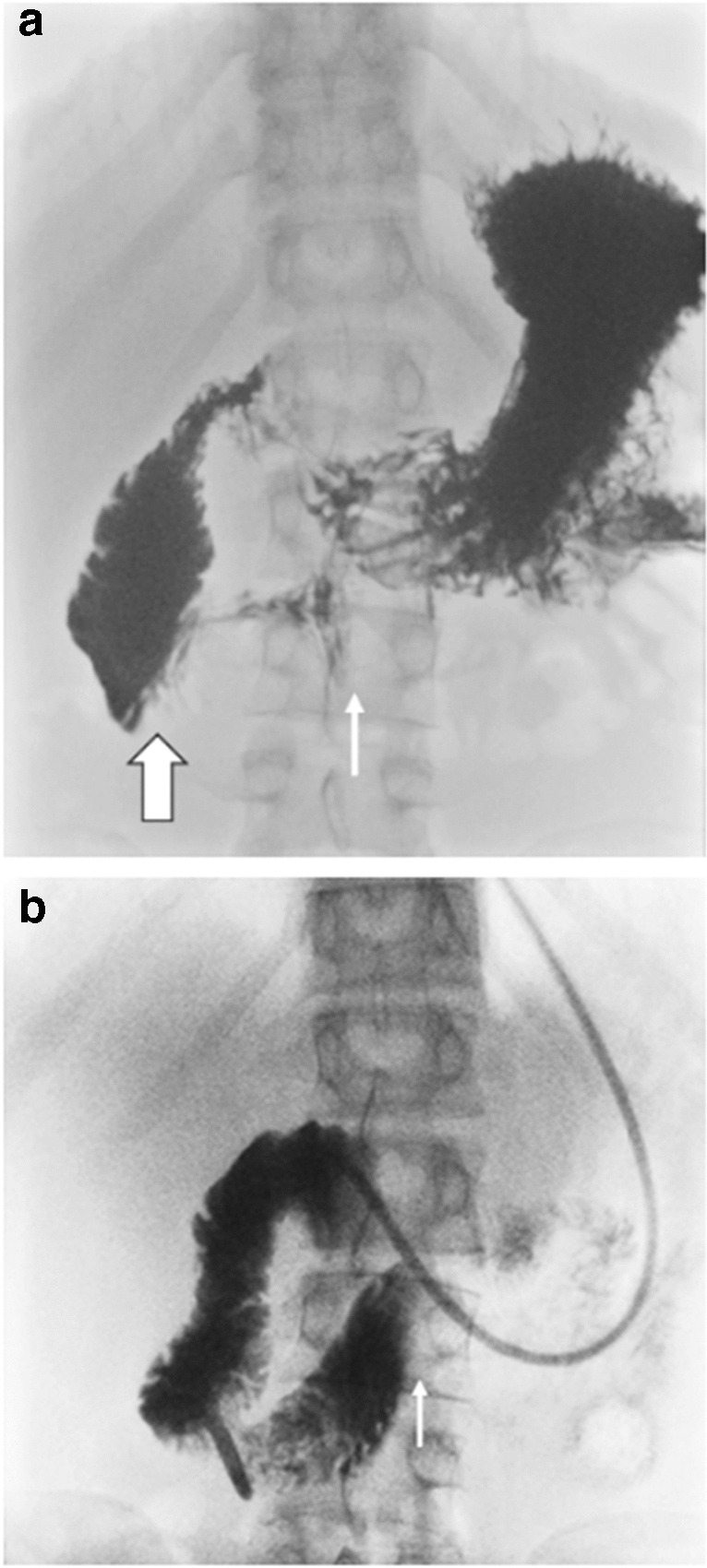
Upper GI contrast study images of a 17-year-old male presenting with weight loss, vomiting, and diagnosis of superior mesenteric artery syndrome. **a** Transient hold-up of contrast both at the inferior duodenal flexure with a sharp oblique vertical medial contour (*thick arrow)* and in the midline (*thin arrow*) on the upper GI contrast study with proximal duodenal dilatation. **b** On follow-up, a nasoduodenal tube was inserted and contrast was injected into the proximal aspect of the 3^rd^ part of the duodenum, just distal to the inferior duodenal flexure. The contrast hold-up persisted in the midline with a sharp vertical contour (*arrow*) in keeping with superior mesenteric artery syndrome. The patient was treated with a high-calorie diet and was discharged with symptomatic improvement

**Fig. 4:**
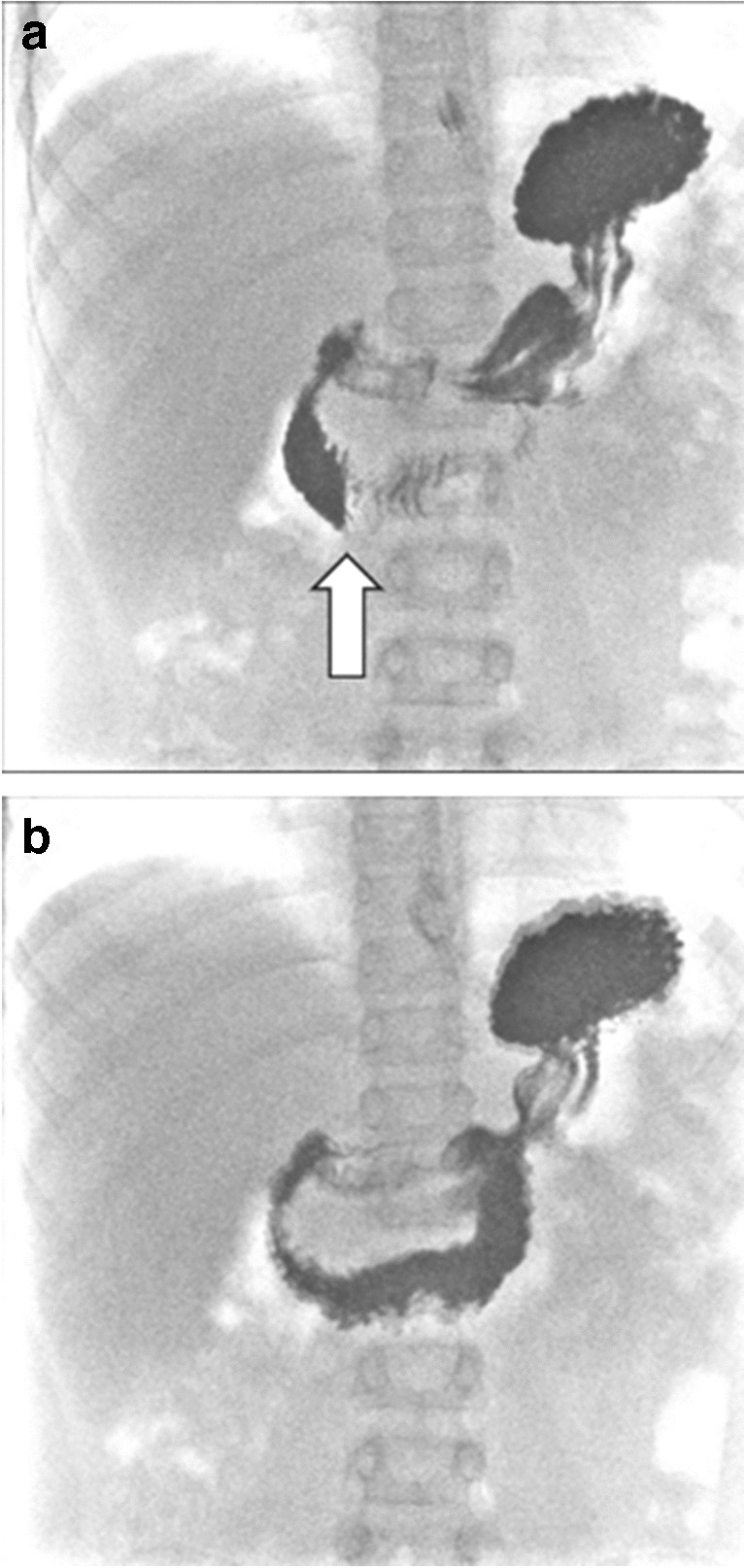
Upper GI contrast study images of a 10-year-old male to evaluate for malrotation. **a** There is minimal transient hold-up of contrast at the inferior duodenal flexure with a sharp vertical medial contour (*arrow*) and no proximal duodenal dilatation. **b** Almost immediate subsequent passage of contrast is seen through the 3^rd^ part of the duodenum distal to the duodenojejunal junction. The patient was discharged home following exclusion of malrotation

There were 5 patients who had a clinical diagnosis of superior mesenteric artery syndrome which was identified in their patient notes (body mass index range 16.8–20.6 kg/m^2^, one patient had scoliosis as a comorbidity). None of the patients with a clinical diagnosis superior mesenteric artery syndrome met our diagnostic criteria for superior mesenteric artery syndrome. All five patients had a right-of midline duodenal impression and one of these patients also had a mid-to-left of midline compression (Fig. 3). The patient with both a right-of-midline impression and a mid-to-left of midline compression did not have a proximally dilated duodenum or “to-and-fro” contrast motion. The remaining four patients did have “to-and-fro” contrast motion, and two of them also had proximal dilation of the duodenum (Fig. 4) suggesting duodenal obstruction with a superior mesenteric artery-like syndrome.

## Discussion

Radiologists should be aware of the normal variant of a right-of-midline impression, in isolation, on the D3 part of the duodenum caused by the IVC [5]. When describing this right of midline finding in the absence of proximal dilation and “to-and-fro” motion, we recommend using the term “impression” rather than duodenal compression as this finding is due to normal duodenal/IVC anatomy and not necessarily a true compression. We also believe that ensuring multiple radiographic signs, not just a mid-to-left of midline compression, is important in suggesting a diagnosis of superior mesenteric artery syndrome [5]. The three radiographic signs that should be used in conjunction with each other on upper GI contrast studies to increase the specificity of diagnosing superior mesenteric artery syndrome include a true mid-to-left of midline duodenal compression, dilated proximal duodenum, and “to-and-fro” motion of contrast flow in the proximal duodenum.

In our sample, only 3 cases (0.6%) displayed all three of our radiologic diagnostic signs, similar to previously published superior mesenteric artery syndrome incidence [40]. More notably, 240 out of 538 studies demonstrated a right-of-midline duodenal impression. This likely represents a normal anatomical finding in the absence of other diagnostic criteria such as proximal duodenal dilation or persistent contrast hold-up (Fig. 4). As mentioned previously, there are multiple cases in the literature where a vertical duodenal impression to the right of the vertebral midline is used in describing/diagnosing superior mesenteric artery syndrome [5, 10–19] which we feel has resulted in the over diagnosis of superior mesenteric artery syndrome. Our findings are consistent with Hines et al. (1984), suggesting that true superior mesenteric artery syndrome is a rare entity [5]. If the duodenal impression is to the right-of-midline, and is associated with signs of partial obstruction (i.e., proximal dilation and “to-and-fro” peristalsis), this should be diagnosed as “superior mesentery artery-like syndrome” as described by Dross et al. (2019) [41]. It has been hypothesized that right-of-midline impression may be caused by the middle colic artery and in extremely rare instances a “superior mesenteric artery-like syndrome” could also be caused by other vascular structures such as the gonadal vein, ileocolic vein, and ileocolic artery [3, 41–43]. Anatomically, it is expected that the duodenal compression associated with superior mesenteric artery syndrome would be found along the midline [14, 33] as the superior mesenteric artery is known to come off of the abdominal aorta at the level of L1 and travel over the D3 part as it supplies blood to the jejunum, ileum, right colon, and usually the transverse colon [9]. Therefore, a diagnosis of superior mesenteric artery syndrome is not supported by impression found to the right of the vertebral bodies, but only by a compression to the mid-to-left of the spine [24, 33, 44]. For both superior mesenteric artery syndrome and “superior mesentery artery-like syndrome,” it is important to ensure the clinical context is appropriate.

**Fig. 5:**
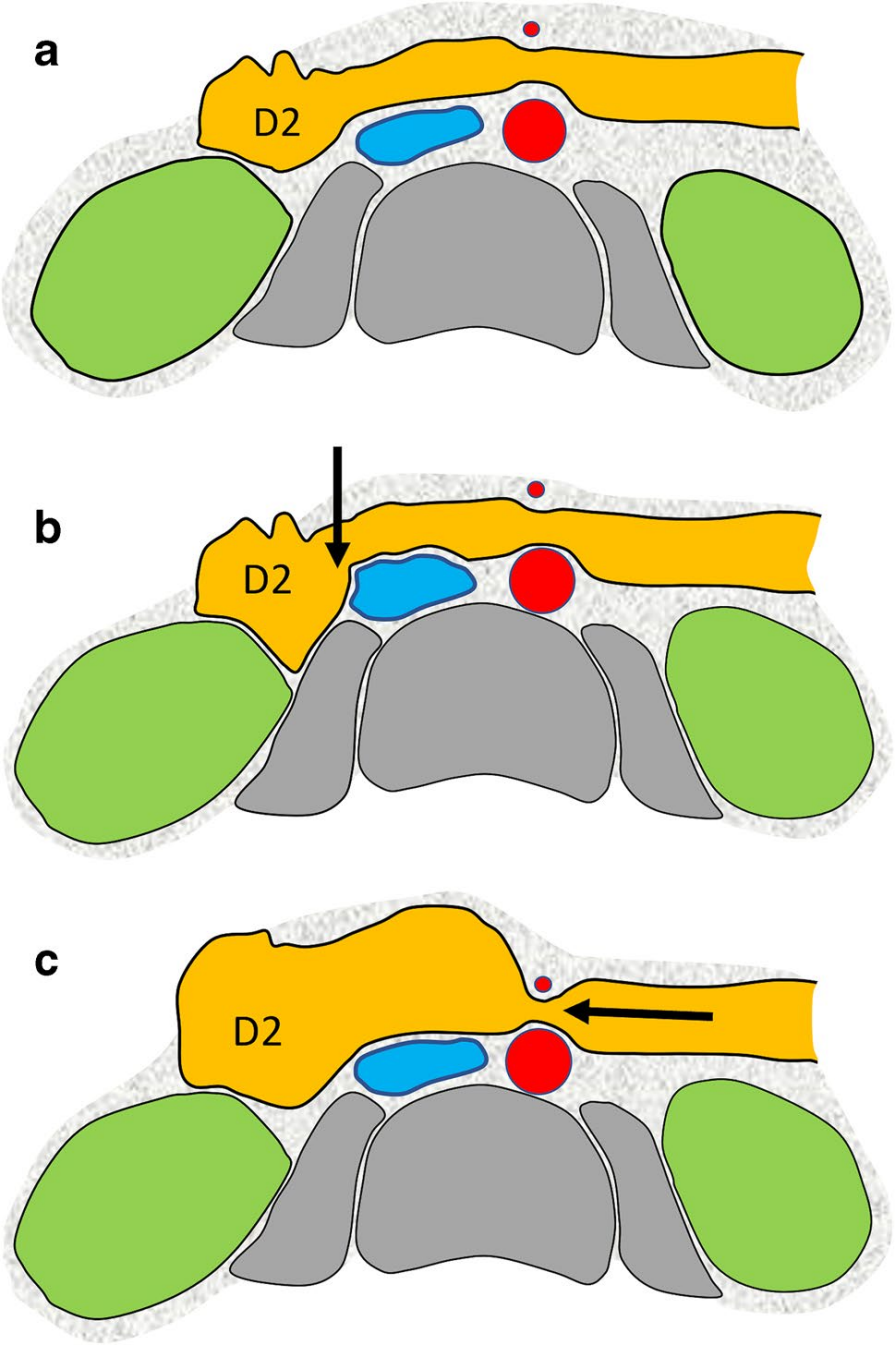
Schematic diagram of the superior mesenteric artery. **a** Normal anatomy of the IVC and the duodenum. **b** Normal anatomical variance showing posterior duodenal relationship to the IVC (*arrow*) resulting in right-of-midline prominent duodenal compression on upper GI studies as outlined in this paper. **c** Superior mesenteric artery syndrome showing significant compression on the 3^rd^ part of the duodenum between the aorta and superior mesenteric artery. D2, second part of the duodenum; blue, IVC; red circles, aorta and superior mesenteric artery; green, kidneys; gray, spine and psoas muscles

**Fig. 6:**
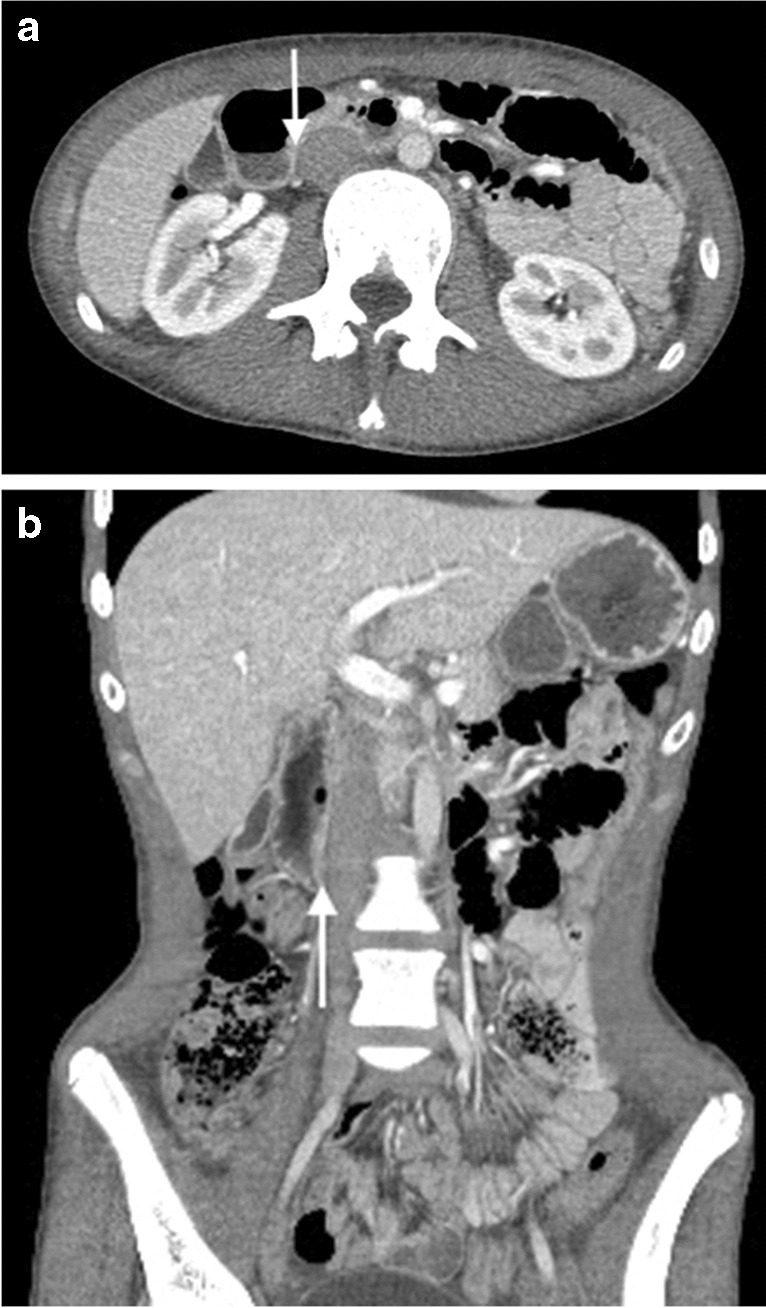
Contrast-enhanced CT images of a 15-year-old female presenting with weight loss, reflux, and dysphagia. **a** Axial image of the duodenum (*star*) having a posterior relationship to the IVC (*I*) creating the right-of-midline duodenal compression (*arrow*). **b** Coronal image of the duodenum (*star*) positioned to the right of the IVC (*I*) creating the right-of-midline duodenal impression (*arrow*) on upper GI contrast studies. The patient was diagnosed with an eating disorder and discharged home with support and no diagnosis of superior mesenteric artery syndrome was made

The right-of-midline impression may be more common in patients with decreased retroperitoneal fat (Figs. 5 and 6), where the posterior portion of this duodenal segment extends past the posterior aspect of the IVC. When contrast flows through this area on upper GI contrast studies (particularly in the supine position) into the D3 part, it likely creates an apparent holdup in the passage of contrast, representing an artificial filling defect of the dependent D2 part along the right lateral wall of the IVC. However, the peristaltic waves of the duodenum and increasing volume of contrast in the D2 part are able to relatively quickly overcome this anatomical hurdle along the lateral margin of the IVC. In the presented analysis, the right-of-midline impression was found to be mainly transient; there were only 15 of 240 cases where contrast hold-up at this point lasted longer than a couple of minutes. With regard to a true definition of what constitutes “persistent” hold-up of contrast within the duodenum, this is difficult as the maximum time a patient was scanned in our study was 30 min. In our experience, any cases of “persistent” contrast hold-up was defined as lasting up to 10–20 min.

There are limitations to our study. None of our limited sample of patients clinically diagnosed with superior mesenteric artery syndrome underwent surgery to confirm a true diagnosis of superior mesenteric artery syndrome. Furthermore, there was a small number of patients who were diagnosed with clinical superior mesenteric artery syndrome, and none of these patients had all three diagnostic criteria which we have proposed. In fact, some of these patients had a right-sided impression without proximal dilation or to-and-fro motion of contrast. Another limitation is that the “persistent” delays in contrast mentioned in our included studies generally ranged from 15 to 30 min, for practical purposes. In our study, we reviewed static images and clinical reports and evaluation of to-and-fro contrast motion is best done in real-time or by evaluating cine clips.

In conclusion, we have shown that a transient right-sided duodenal impression can be a normal finding and should not be confused for the diagnosis of superior mesenteric artery syndrome when identified in isolation. Radiologists and pediatricians should also be conscious of using multiple radiologic and clinical diagnostic criteria when diagnosing superior mesenteric artery syndrome.

## Data Availability

The datasets generated and analyzed during the current study are available from the corresponding author upon request.
